# Human papillomavirus (HPV) and Epstein-Barr virus (EBV) in keratinizing versus non- keratinizing squamous cell carcinoma of the oropharynx

**DOI:** 10.1186/s13027-018-0205-6

**Published:** 2018-11-09

**Authors:** Francesco Broccolo, Giulia Ciccarese, Agostino Rossi, Luca Anselmi, Francesco Drago, Antonio Toniolo

**Affiliations:** 1 Department of Medicine and Surgery (School of Medicine), University of Milano-Bicocca, Monza, Italy; 2 DISSAL Department of Dermatology, IRCCS A.O.U. San Martino-IST, Genoa, Italy; 3 Laboratory of Medical Microbiology, Department of Biotechnology and Life Sciences, University of Insubria, Varese, Italy; 4 Department of Pathology, ASL 3 Genovese, Genoa, Sampierdarena Italy; 5 Laboratory of Medical Microbiology, University of Milano-Bicocca, Via Cadore 48, 20900 Monza, Italy

**Keywords:** EBV, HPV, Oral, Oropharyngeal, Cancer, HPV-induced p16^INK4A^ immunohistochemistry, Co-infection

## Abstract

**Background:**

Oral and oropharyngeal squamous cell carcinomas (OSCC and OPSCC) represent the majority of head and neck squamous cell carcinomas (HNSCC). Human papillomavirus (HPV) is an important etiologic factor together with Epstein-Barr virus (EBV). Little is known on the prevalence of major herpesviruses [EBV, cytomegalovirus (CMV) and HHV-6, − 7 and − 8] in HNSCCs.

**Methods:**

Fifty-one formalin-fixed paraffin-embedded (FFPE) tissue samples taken at surgery (40 oropharyngeal, 11 oral) were analyzed for 40 HPV genotypes (20 high-risk types), EBV, CMV, HHV-6, − 7 and − 8 by quantitative PCR. Expression of the HPV-induced p16^INK4A^ protein was also investigated by immunohistochemistry (IHC).

**Results:**

In SCC, the prevalence of EBV was significantly higher compared to that of HPV (EBV 51% vs. HPV 19.5%; *P* = 0.005). HPV infection was found in 25% of OPSCC and in none of the OSCC; conversely, higher prevalence of EBV was found in OSCC (72.7%). HPV and EBV co-infection was detected only in 4 (10%) OPSCC. CMV was detected in only two cases, whereas HHV-6, − 7 and − 8 resulted negative. The prevalence of HPV but no EBV was associated with the non-keratinizing SCC type (NKSCC) compared to the keratinizing SCC type (KSCC)(HPV-DNA *P* < 0.005; EBV = 0.054).

**Conclusions:**

Single HPV or EBV positivity was higher in OSCC than in OPSCC. Other potentially oncogenic herpesvirus types were minimally or not represented.

## Background

Head and neck squamous cell carcinomas (HNSCC) account for approximately 5% of all cancers and are a serious public health problem worldwide [1]. HNSCC comprise a large group of tumors classified as oropharyngeal squamous cell carcinomas (OPSCC) and oral squamous cell carcinomas (OSCC). HNSCC is etiologically linked to tobacco and/or alcohol consumption [2]. Although chemical risk factors have been widely studied, research revealed that human papillomaviruses (HPV) do play an important role in a subset of HNSCC [3]. Currently, 20–30% of patients with OPSCC do not have the traditional risk factors of smoking and alcohol use and HPV appears as the major driver of malignant transformation [4]. Case-control studies reported a consistent association of HPV infection with HNSCC, particularly for OPSCC [2, 5, 6]. In contrast, the association of HPV with oral and laryngeal cancers has been inconsistent, possibly due to tumor misclassification and the influence of confounding factors like tobacco and alcohol consumption [4]. Exposure to HPV in the head and neck region is associated with sexual behaviors that increase the risk of HNSCC [7]. The association between HPV infection and head and neck carcinogenesis is based on histological similarities between the anogenital epithelium and the squamous epithelium of the head and neck region, as well as on the detection of most common oncogenic HPV genotypes in both cervical cancer and HNSCC [8]. Based on epidemiologic observations, 12.8 to 59.9% HNSCCs are associated with HPV infection [9]. In Europe, the estimated prevalence of HPV in these tumors is approximately 41% [10]. Limited data are available for Italy regarding HPV detection in HNSCCs, with prevalence ranging from 10 to 46% [11, 12].

The Epstein-Barr virus (EBV) has long been related to nasopharyngeal carcinoma [13]. A recent meta-analysis show a significant association between EBV and OSCCs [14] though there are studies not reporting a significant association [15]. Some members of Herpesviridae family (EBV and HHV-8) are recognized as carcinogens. Other herpesviruses such as cytomegalovirus (CMV) and HHV-6 and -7 may be associated with a malignant phenotype, but their carcinogenic role remains unclear [16]. Persistent infection with EBV has been linked to the development of malignancies including HPV-associated oral carcinoma. However, the possible role of EBV and other herpesviruses in HPV-associated oral cancers is still poorly understood. The majority of studies were based on HPV-DNA markers and failed to investigate the expression of P16^INK4a^ a surrogate marker of oncogenic HPV infection. P16^INK4a^ is a tumor suppressor protein that is downregulated in many tumor types [17], but overexpressed in HPV-related tumors [18]. Very little is known on the possible co-carcinogenic role of EBV and other herpesviruses in HNSCC. We thus evaluated the prevalence of the above agents and their possible co-infection rate in a series of Italian SCC cases.

## Material and methods

### Tissue samples

Archived formalin-fixed, paraffin-embedded (FFPE) tissue blocks were collected for 51 SCC cases. FFPE sections were obtained from the archive of the Pathology Department. Tissue specimens were obtained from patients with HNSCC (oral cavity and oropharynx) before initiation of radiotherapy, while samples of laryngeal HNSCC were taken at surgery. Eligible cases included newly diagnosed primary HNSCC. At the time of sampling, patients had not received antitumor therapy. The study was approved by the Ethics Committee of IRCCS San Martino IST (Genoa, Italy). As archived tissue was used, no written consent was required.

5 × 10 μm sections were removed from the blocks were deparaffinized with xylene and rehydrated through a graded series of distilled water-ethanol solutions. QIAamp DNA FFPE Tissue Kit (QIAGEN, Hilden, Germany) was used for DNA extraction. DNA was quantified with a NanoDrop 3300 fluorospectrometer using the RiboGreen dye diluted in TE buffer (Thermo Fisher Scientific, Monza, Italy).

### Detection of human herpesviruses (HHV) and HPV genotypes

Tissue sections were retrospectively analyzed for EBV and other HHV (CMV, HHV-6, − 7, − 8) by quantitative real-time PCR assays [19]. HPV detection and genotyping was performed using a multiplex real-time PCRs based on hybridization-fluorescence detection of amplified products (PANA RealTyperTM HPV assay – Panagene; DBA Italia, Milano, Italy). The assay was performed for detection and differentiation of 20 high-risk HPV types (16, 18, 26, 31, 33, 35, 39, 45, 51, 52, 53, 56, 58, 59, 66, 68, 69, 70, 73, and 82), two low-risk genotypes (6 and 11) from clinical specimens. Eighteen genotypes (30, 32, 34, 40, 42, 43, 44, 54, 55, 61, 62, 67, 74, 81, 83, 84, 87, and 90) are detected, but not typed.

### **Immunohistochemistry (IHC) for** p16^INK4A^

Immunoperoxidase staining was done on 4 μm sections of FFPE tissue using the LSAB2 horseradish peroxidase system (DAKO, Carpentaria, CA). Antigen retrieval was done by microwave heating for 10 min in 10 mM citrate buffer (pH 6.0). A p16^INK4A^ monoclonal antibody (Novocastra-Leica, Buccinasco, Italy) was used at 1:50 dilution. Cases were classified as either positive (with cut-off value of stained nuclei > 40%) or negative.

### Statistical analysis

Statistical analysis was performed for virus positivity, p16^INK4A^ positivity, clinical and demographic data using the Prism GraphPad software (San Diego, CA). Statistical significance was defined as *p* < 0.05.

## Results

The study included 51 patients, 40 with oropharyngeal cancer (OPSCC: 5 in pharinge, 27 in larynge, 5 in vocal cord, 3 in base of tongue) and 11 with oral cancer (OSCC: 7 in floor of the mouth, 3 in buccal mucose, 1 in tongue). Demographic and clinical data were also recovered. Mean age was 65 years (standard deviation ±14 years) ranging from 43 to 94 years. Males (69%) with smoking (70%) and alcohol abuse (60%) problems were prevailing in the series. All cases resulted negative for HHV-6, − 7 and − 8. CMV was detected in only two cases (mobile tongue SCC and epiglottis SCC).

Overall, of the 51 patients, 10 cases (19.6%) were positive for HPV (9 HPV-16 and 1 HPV-45) and 12 (23.5) for p16^INK4A^.. Two patients had p16^INK4A^positive and negative HPV PCR.

The prevalence of HPV and EBV, according to SCC primary site and hystotype and is presented in Table 1. In particular, the prevalence of EBV resulted significantly higher compared to that of HPV (EBV 51% vs. HPV 19.6%; *P* = 0.05). Regarding the subsites of the head and neck region, HPV infection was found in 25% of OPSCC and in none of the OSCC. Conversely, EBV infection was found in 72.7% of OSCC and 45%.of OPSCC cancer cases (Table 1) HPV and EBV co-infection was detected in 4 (10%) OPSCC. The prevalence of HPV but no EBV was associated with the non-keratinizing SCC type (NKSCC) compared to the keratinizing SCC type (KSCC)(HPV-DNA *P* < 0.005; EBV = 0.054).

**Table 1:** Expression of HPV-induced p16^INK4A^ and prevalence of HPV and EBV in squamous cell carcinoma according to primary tumor site and histotype

	HPV-induced p16^INK4A^	HPV^+^ (%)	EBV^+^ (%)	Co-infection HPV-EBV (%)
Primary site (No.)				
OPSCC (40)	11 (27.5)	10 (25)	18 (45)	4 (10)
OSCC (11)	1 (9.1)	–	8 (72.7)	
Histotype (No.)				
NKSCC (26)	11 (42.3)	9 (34.6)	16 (61.5)	4 (15.4)
KSCC (25)	1 (4)	1 (4)	10 (38.5)	

*KSCC* keratinizing squamous cell carcinoma, *NKSCC* nonkeratinizing squamous cell carcinoma. HPV p16 detected by immunohistochemistry (IHC)

**Fig. 1:**
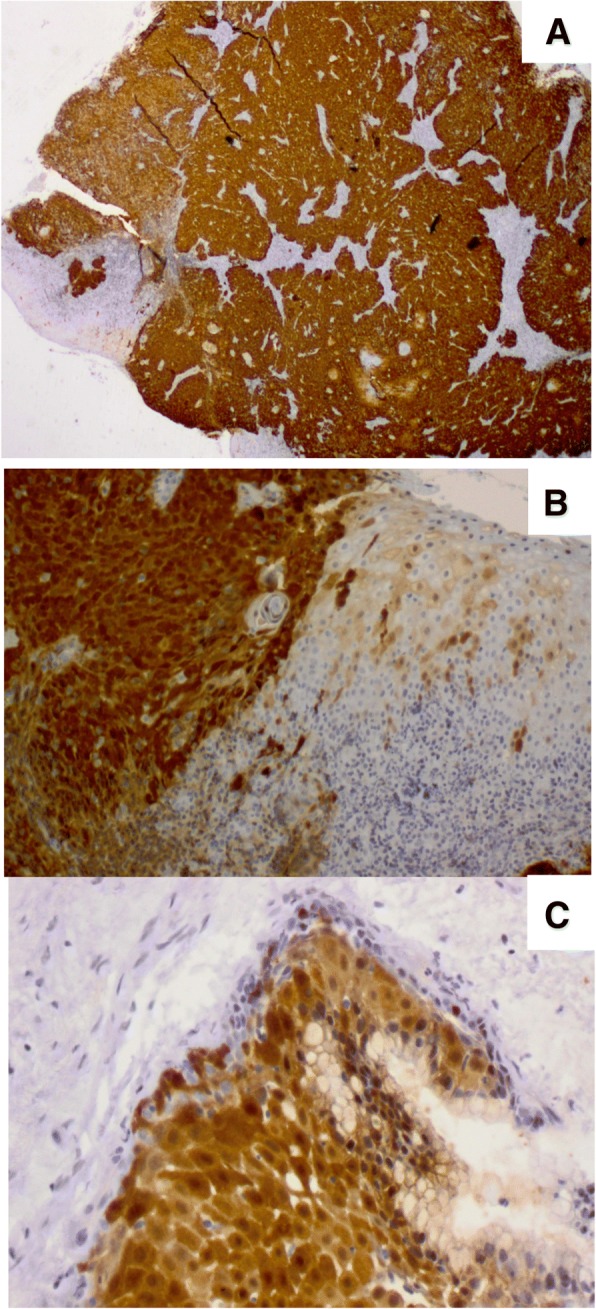
Expression of HPV-induced p16INK4A in squamous cell carcinoma (SCC). (**a**-**b**) p16^INK4A^-positive section of non-keratinizing SCC at the base of tongue (2x and 10X magnification); (**c**) p16^INK4A^-positivity in laryngeal SCC (10X magnification)

Of note, all 9 NKSCC cases HPV positive were also p16^INK4A^ -positive. In contrast, only 1/15 KSCC cases was HPV positive and also p16^INK4A^ -positive. The data indicate that NKSCC cases are more likey p16^INK4A^ - and HPV- positive than KSCC (*P* = 0.0001 and *P* < 0.05, respectively). In Fig. 1 is showed HPV-induced p16^INK4A^-positive NK SCC at the base of tongue and in laryngeal. For EBV, although there was no difference in histopathological type, NKSCC cases were also more frequently EBV-positive compared to KSCC cases (data not shown).

## Discussion

We explored the prevalence of HPV and potentially oncogenic herpesviruses (HHVs) in archival tissue of SCC cases from different anatomic sites. This is the first study from Italy reporting on the HPV and EBV co-infection in cancers.

The overall HPV prevalence in HNSCC was low for OPSCC (25%) and absent in OSCC compared to the reported global prevalence of HPV in the same types of cancer (45.8% OPSCC, 24.2% OSCC, respectively) [9, 10, 18] but in accordance with one Italian study (OPSCC 27%) [11].

Among the 12 HPV types defined as carcinogenic in cervical carcinoma by the International Agency for Research on Cancer (types 16, 18, 31, 33, 35, 39, 45, 51, 52, 56, 58 and 59), only type 16 appears to play an oncogenic role in OPSCC [9]. Among HPV-positive OPSCC cases, HPV16 was the most prevalent genotype (9 of 10 cases)., HPV-45 DNA was detected in 1 case suggesting an oncogenic role of this genotype in OPSCC as well as cervical carcinoma.

The detection of HPV DNA alone is not sufficient to determine tumor causality. For cervical carcinoma and for oropharyngeal and laryngeal cancer the co-expression of several markers (alone or in combination) is required to discriminate HPV-driven from non-HPV-driven cases [20].

The p16^INK4a^ protein is considered a surrogate marker of HPV infection in SCC of the cervix and head and neck, as well as in HPV-associated anogenital cancer [21]. In HNSCC, p16^INK4a^ appears as a reliable marker in tumors arising from the oropharynx, larynx, and oral cavity [21, 22]. A portion of oropharyngeal tumors show upregulated p16^INK4a^, but lack HPV positivity.

In our series, infection with high-risk HPV genotypes was not significantly associated with oral cancers. This is in line with what reported [4]. In contrast, a strong association of high-risk HPV genotypes with oral cancer has been observed by others [3].

We classified SCC histotypes into two groups: nonkeratinizing and keratinizing SCC. In the investigated series, NKSCC was strongly associated with high-risk HPV genotypes and p16^INK4A^ positivity (69% and 100%, respectively) compared to KSCC (8% and 36%, respectively). Of interest is the frequent detection of HPV and EBV co-infection in oral and oropharyngeal cancer among Italian patients. HPV infection is considered a sexually-transmitted infection, while EBV is mostly transmitted through saliva [23]. Both agents are capable of establishing long-term infection in epithelial cells. A variety of stimuli do play a role in activating viral genome expression [23]. Chemicals and sexual behaviors can play a role. The results of our study confirm the low prevalence of HPV- driven HNSCC in Italy, in line with data from other countries of southern Europe. HPV16 appears to play a role mostly in the oropharynx, while EBV is more prevalent in the oral cavity cancer. Thus, current HPV vaccines [24] may be expected to prevent part of head and neck cancer, but an EBV vaccine remains badly needed [23].

## Abbreviations

**CMV**: cytomegalovirus
EBV: Epstein-Barr virus
FFPE: formalin-fixed paraffin-embedded
HHVs: human herpesviruses
HNSCC: head and neck squamous cell carcinoma
HPV: Human Papilloma Virus
OPSCC: oropharyngeal squamous cell carcinoma
OSCC: oral squamous cell carcinoma
QPCR: quantitative Real-time PCR
